# The Air We Breathe

**Published:** 1880-04

**Authors:** Ben. H. Pratt


### THE BISTOURY.

Vol. XVI. ELMIRA, N. Y., APRIL, 1880. No. 1.

### Contributions.

### THE AIR WE BREATHE.

BEN. H. PRATT.

Half the world is committing slow suicide by poison. We are dying by degrees for the want of pure air. One of the most important functions of the human family and of all animal kind is their breathing. People are not apt to give much attention to an evil they cannot see unless they are apprised of it by a shock. The air we breathe is such a common thing to us, and most of us inhale it with so little of a realizing sense that we are doing so, that we take no note of it. Until one stops to consider that he is breathing—that he is filling his lungs with air and empting them again at the average rate of fifteen times a minute—or is reminded of his respiration by a sudden interference therewith—the operation goes on without his worrying over it or even having a knowledge of it. If, now, the atmosphere which enters into and is exhaled from the lungs becomes noxious so imperceptibly that we are not made suddenly aware of the fact, we are very likely to be kept in ignorance of that fact until something unusual happens in the way of a sensation, and even then we are liable to attribute the sensation to something else. The insidiousness of the change is its danger, and harm often, indeed almost generally, comes to us before we are aware that harm is lurking near us.

If one could, by some process of introspection not yet discovered, look into one of his lungs while it is performing its normal functions, and could observe the delicacy of its structure, the perfection of its organization and the wonderfulness of its operation, he would be far more careful of the quality of the air which enters it and permeates the ramifications of its complicated structure. If one have a good watch, the derangement of the mechanism of which can at most cause him a mere inconvenience and a loss of a few dollars, he nevertheless uses extreme caution when he opens it and exposes it to the air, lest some minute speck borne on the wind, or some mere fleck floating in the air get between the tiny cogs of its wheels, or a particle of moisture enter and rust some of its delicate pinions. Yet the mechanism of the daintiest and costliest timepiece that human ingenuity and skill ever devised and constructed is a clumsy and a botchy thing compared with that marvel of the anatomical structure we call a lung. Yet one takes little thought of the latter and takes much thought of the former, simply because he sees and knows the delicacy of the one and not of the other. If one would but take the pains to call upon his butcher and get him to exhibit to and inflate for him a fresh lung taken from the recently slain body of a sheep or other animal, one might glean from this some faint idea of the extreme delicacy of structure and of the singular sensitiveness of the pair of bellows one continually carries about with him and which is, without any voluntary effort on his part, continually at work vitalizing the blood by bringing to it its vivifying principle and removing from it all that which would soon render it incapable of coursing the arteries and veins of the system. It would also make him more careful how he uses his lungs and to what he subjects them, and teach him how easily they may become unfitted for their work and finally of what vital importance they are in the economy of life. Our respiration is our life. Stop or hinder the action of the lungs, and the whole system suffers a prostration in a direct ratio with the interposing action. Upon the perfect action of the lungs and the purity of the air inhaled depend the condition of the blood, and upon this depends the health of the containing body. It is unnecessary to enter into the process by which the circulation of the blood, from the heart, through the arteries, into the veins, and thence to the lungs, bearing its burden of impurities gathered in its course to the latter organs to be revitalized by the air with which they are inflated and thence restored to the heart as pure as before it had done all this scavenging; I say it is unnecessary to explain all this, but it is necessary to know that unless the air that enters the lungs be pure the operation can not be perfect, and every round the blood makes, it becomes more and more impure until it no longer does its scavenging, and the system becomes poisoned in every atom of its structure.

All have felt the stupidity that arises from confinement in a close room for many hours at a time, and all have experienced the glad exhilaration that comes of a few draughts of pure out-door air after such confinement. If the apartment which we have been occupying has been occupied by others as well, then the quickness with which the sensation of drowsiness or stupidity comes is in proportion to the number of those with us, other things being equal. All have felt that peculiar drowsiness which often comes upon them in church or in other places where people are wont to gather in any numbers for any length of time. The dullness comes upon us so gradually that we are seldom conscious that we are anything save inclined to sleepiness, and then we lay it to anything but the real cause. We attribute it to the dull sermon, or to indigestion, or to the lack of our usual exercise, or to a cold caught somewhere, or to walking in the wind, or to sitting up a little later than usual the night before, or to getting up a little earlier than we ought, or to being kept awake by the baby, or to something of these sorts. Nothing of the kind. In nine cases out of ten the tendency to sleep is owing to an insufficiency of oxygen in the air we are breathing and a corresponding excess of carbon. Hundreds of pairs of lungs have been at work in that room—constantly at work—using up the oxygen to vitalize the blood, and giving back the carbon generated by that eternal combustion going on within us—extracting all the vital principle and loading the air with poison, but which we keep pulling into our lungs and thus taking poison at every breath as surely as though we drank it into our stomachs. We reinhale what we have thrown off, it gets back into the blood, that fluid becomes laden down with more weight at each circuit, and by and by it can not flow with its usual alacrity, grows sluggish, as you might readily discover were you to note the slowing and the fulling of the pulse and the distention of the veins. The vitiated blood passes to the brain, that organ becomes dull and refuses to do its normal work because its nourishment is cut off in a measure. It takes a rest—must have it—ceases to act—we go to sleep unless strenuous efforts to the contrary are made. We may pinch and prick ourselves into a semi-conscious state and get through without nodding, but the most brilliant sermon ever preached can not keep a poisoned brain in action. Such sensations are as surely indications of a beginning of death as are the first symptoms of somnolence after a dose of laudanum, and if we remained long enough under such circumstances our case must prove fatal. But as we still abide upon that sphere

> Where congregations do break up
> And Sabbaths have an end, we get an opportunity to administer the antidote, fresh air, before the vital spark has forever fled, and a few inhalations restore us to our wonted animation. But it is folly to pretend that we can keep this thing up from day to day, or from week to week, without doing permanent harm. At each subsequent exposure to the poisonous influences of impure air we are less able to endure it, and by and by its effects are visible in a weakened organ, the lungs, the heart, the stomach, or all of these; for their interdependence is one of the grandest mysteries of the anatomical economy. Air deficient in oxygen is found in every room that is closed if it be occupied by a living breathing being. There are those who spend three fourths of their lives in such rooms and still wonder what ails them. There are those who have become invalids too weak to endure the stimulant of natural out-door air, because their organs of respiration have become enfeebled by their long habit of using a diluted article. Air seems to be the terror of many. A door a crack open, a window sash a trifle loose in its run-way, will cause an alarm, not because of the cold that may enter, but because, somehow or other, people have a dread of out-door air. Many while out of doors are afraid to talk much lest they "get some air on their lungs," and others keep a handkerchief or a muff or a glove to the mouth in cold weather lest they "catch cold on their lungs." It is evident that air was made before lungs were, and it is altogether likely that lungs were made to match the air. Some don't believe the Almighty knew much when he settled the relation of things here below—at least they act as though they had no confidence in His adaptations.

As to the relative wholesomeness of cold and warm air there is a variety of hobbies. There are those who think they can not exist in an indoor temperature lower than seventy degrees during their waking hours, yet fully as firm are they in their belief that they would smother at night in a temperature higher than forty in the winter time. They do not exchange their day-lungs for a night pair when they retire; yet they proceed upon the principle that such is the case. Others go still further, and contend that the windows of their dormitories must be wide open the year around, be the weather what it may. Others still will spend the day in rooms comfortably heated, and retire between sheets which chill them to the bone as effectually as though they plunged into an ice-bath. Again there are those who, reasoning upon the principle that, sleeping or waking, the temperature of their apartments should be uniform, sleep with closed windows and doors, and during the latter half of the night breathe an atmosphere heavily laden with rank poison.

It is reasonable that during the time in which we are putting forth little or no muscular effort the temperature should be higher than when we exert ourselves, and therefore it is that one may sit comfortably where he could not work comfortably, and one may walk, ride or work comfortably in a temperature so low that it would be dangerous to sit in. It is also reasonable to conclude that in proportion as we are active, in the same proportion the temperature should be lowered, and vice versa; hence our sleeping rooms should be the warmest of our apartments, our work rooms the coolest and our sitting rooms of a mean temperature between these. To go from a warm sitting room into a cold sleeping room is one of the greatest as well as one of the commonest mistakes we encounter. Few can stand the repeated shock for any length of time.

Another fallacy is the notion that warm air is necessarily foul, and cold air pure. It is often the reverse of this. Rooms may be warm and at the same time kept pure by means of a simple process of ventilation. Ventilation is necessary to every occupied apartment, as it has been shown that air is soon vitiated by exhalations from the lungs, and this vitiation is intensified by the various processes in vogue of heating and lighting our dwellings. Combustion, even by a single lamp or gas jet, takes up oxygen, the life of the air, and evolves carbon, the poisoning principle. That without systematic ventilation, by which the within air is driven forth and replaced by that from without, children get to be men and women, and these live to old age, is accounted for by the fact that our dwellings are not, fortunately for us, air tight. There are in the best constructed dwellings, cracks and crevices through which vitiated air is kept from becoming fatally noxious. Beside these are the occasional opening and closing of doors, and a consequent in-come of pure and out-go of impure air. Were this not so, few would live, in this age, to manhood and womanhood. The poorer the house, the better the natural ventilation; and hence it is that our better classes of dwellings, where the door fittings and the window fittings are exact, the floors filled in and covered with air-excluding carpets, and paper or fresco or other adornments make hermetrically sealed boxes of living apartments, it becomes essential to introduce a systematic plan of ventilation, to the study of which the minds of architects are now being very seriously and perplexingly turned. The subject of this article here runs into this channel, but it is too wide and deep to enter upon at this time, and I will wait for another opportunity to give a few common-sense views upon this subject which is yearly growing a more and more important factor in domestic comfort, if not, indeed in the preservation of life. I have at this time merely glanced at some of the mistaken notions entertained by people otherwise intelligent, with the design of suggesting the application of the same common sense to this subject that they apply to most other things. It does seem as though there is a wider difference of opinion concerning the effect of pure air upon individual health, with less reason for the holding of such opinions, then upon any other question. If there is one subject upon which people seem utterly uncertain of anything certain, and unable to bring common sense to bear practically upon, it is that of their relation to their surroundings.

What is most necessary to impress upon the generality of people are these points :

Cold air is not necessarily pure air; warm air is not necessarily impure air; the air of any apartment, whether warm or cold, must be impure to a greater or less extent, unless frequently changed, and the extent of its impurity depends upon the number of persons occupying it and the number of stoves and lights within it; and finally, one *may* become conscious of the fouling of the atmosphere of a room, from his sensations, but his sensations are not to be depended upon, and are often attributable to anything but the real cause.

